# Prevalence of new‐onset diabetes following COVID‐19 infection: A systematic review and meta‐analysis

**DOI:** 10.1111/dom.70508

**Published:** 2026-01-28

**Authors:** Jordan N. Keels, Rose D. LaPlante, Christopher S. Lee, Andrew A. Dwyer

**Affiliations:** ^1^ Boston College William F. Connell School of Nursing Chestnut Hill Massachusetts USA; ^2^ P50 Massachusetts General Hospital ‐ Harvard Center for Reproductive Medicine Chestnut Hill Massachusetts USA

**Keywords:** COVID‐19, diabetes, long COVID, new‐onset diabetes, SARS‐CoV‐2

## Abstract

**Aim:**

To estimate the prevalence of new‐onset diabetes in adults (≥ 18 years) following SARS‐CoV‐2 infection.

**Materials and Methods:**

This meta‐analysis includes studies written in English that measured the number of adults (≥ 18 years) diagnosed with diabetes following SARS‐CoV‐2 infection. Studies underwent dual independent review; quality was assessed by using the New Castle Ottawa Scale. A random‐effects meta‐analysis was conducted to obtain the pooled estimate of new‐onset diabetes. To understand the relationship between patient characteristics (age, sex) and study variable (duration of follow‐up), a random effects meta‐regression was used.

**Results:**

A total of 33 articles were retained for analysis. The overall estimated prevalence of new‐onset diabetes (combined T1DM and T2DM or undefined) was 8.33% (95% CI 7.47, 9.18%, z = 19.04, *p* < 0.001; Q = 6791.24, *I* ^2^, 99.68%). The overall estimated prevalence of new‐onset T2DM in COVID‐19 was 8.92% (95% CI 7.88%, 9.96%, z = 16.77, *p* < 0.001; *Q* = 27659.74; *p* < 0.001, *I* ^*2*^ = 99.96%). The overall estimated prevalence of new‐onset T1DM was 0.86% (95% CI 0.0072%, 0.0099%, z = 12.59, *p* < 0.001; *Q* = 9456.28; *p* < 0.001, *I* ^*2*^ = 99.94%). At the study level, there was no significant relationship identified with age, sex, or follow‐up duration.

**Conclusions:**

This systematic review and meta‐analysis revealed a notable increase in T2DM or combined (T1DM, T2DM, or undefined) conditions. As such, it may be important to understand the underlying factors contributing to increased prevalence.

## 1 INTRODUCTION

Diabetes (DM) is one of the most common chronic diseases globally and a major public health concern worldwide.^1^ Importantly, DM is associated with increased morbidity and mortality^2^ with well‐established microvascular (i.e., retinopathy, neuropathy, and nephropathy) and macrovascular complications (i.e., heart failure, cerebrovascular events, myocardial infarction, peripheral vascular disease).^3^, ^4^ The economic cost for diabetes care in 2022 was $412.9 billion dollars, accounting for 1 in 4 health care dollars in the United States (U.S.) and costs are projected to rise over the next 30 years.^5^

New‐onset type 1 or type 2 diabetes mellitus (T1DM, T2DM), following COVID‐19 infection has emerged as a clinically significant area of research on long COVID. Long COVID refers to post‐acute sequalae of COVID‐19 (PASC) affecting both pulmonary and extrapulmonary organ systems.^6^ Several reports point to an increased risk for metabolic disorders (i.e., diabetes) up to 2 years after COVID‐19 infection.^7^

A relationship between COVID‐19 and metabolic dysfunction was identified early in the COVID‐19 pandemic. Early on, obese patients and patients with T2DM were considered to have a higher risk for severe infection and increased mortality.^8^ In addition, corticosteroids were used as a standard of treatment for COVID‐19 infection and are well known to affect metabolic control. Several published studies have reported on the prevalence or incidence of new‐onset diabetes following COVID‐19 infection^9^, ^10^, ^11^, ^12^, ^13^, ^14^, ^15^, ^16^, ^17^, ^18^, ^19^ and a number of mechanisms have been proposed.^19^, ^20^, ^21^ Reported risk factors for developing DM after COVID‐19 infection include male sex, age ≥60 years, individuals who identify as Black, and persons with existing comorbidities including obesity, prediabetes, cardiovascular disease, hypertension, hyperlipidemia, and tobacco use.^22^ Notably, patients with both diabetes and COVID‐19 are at an increased risk of acute respiratory distress syndrome, admission to intensive care, mechanical ventilation, and death.^15^

However, the prevalence and factors influencing new‐onset DM are inconsistently reported across studies, and the phenomenon has yet to be fully elucidated. Previous studies have reported the risk of new‐onset diabetes ranging from 15.53% to 64%.^13^, ^23^, ^24^ As such, the relationship between new‐onset diabetes after COVID‐19 infection remains unclear. We used meta‐analysis to quantitatively synthesise published literature on the prevalence of new‐onset diabetes after COVID‐19 infection. In addition, we used meta‐regression to strengthen our understanding of the relationship between patient characteristics (sex, age) and study variables (duration of follow up).

## 2 METHODS

A systematic review and meta‐analysis of existing literature to examine the prevalence of new‐onset diabetes in individuals with COVID‐19. The review was registered (CRD42024506113) and findings are reported according to the Preferred Reporting Items for Systematic Reviews and Meta‐Analyses (PRISMA) guidelines.^25^

### 2.1 Data sources and search strategy

In collaboration with a research librarian, a systematic search was conducted in PubMed, Web of Science, CINAHL, and Embase, using the following MeSH headings: *diabetes* and either the keyword new‐onset diabetes, newly diagnosed diabetes, diabetes type 1, diabetes type 2, diabetes mellitus, or MeSH heading COVID‐19, long COVID, SARS‐CoV‐2, long COVID syndrome, post‐acute sequelae of COVID‐19, and PASC or MeSH heading *Incidence* or prevalence.

### 2.2 Eligibility criteria

Studies were considered eligible if they met the following criteria: (1) the sample or subsample consisted of new‐onset diabetes patients (2) the prevalence (i.e., *n* or rate with denominator) of new‐onset diabetes in the sample or subsample of COVID‐19 patients was available. Both randomised control trials and cohort studies were considered for inclusion. Studies not published in English were excluded.

### 2.3 Study selection

**FIGURE 1:**
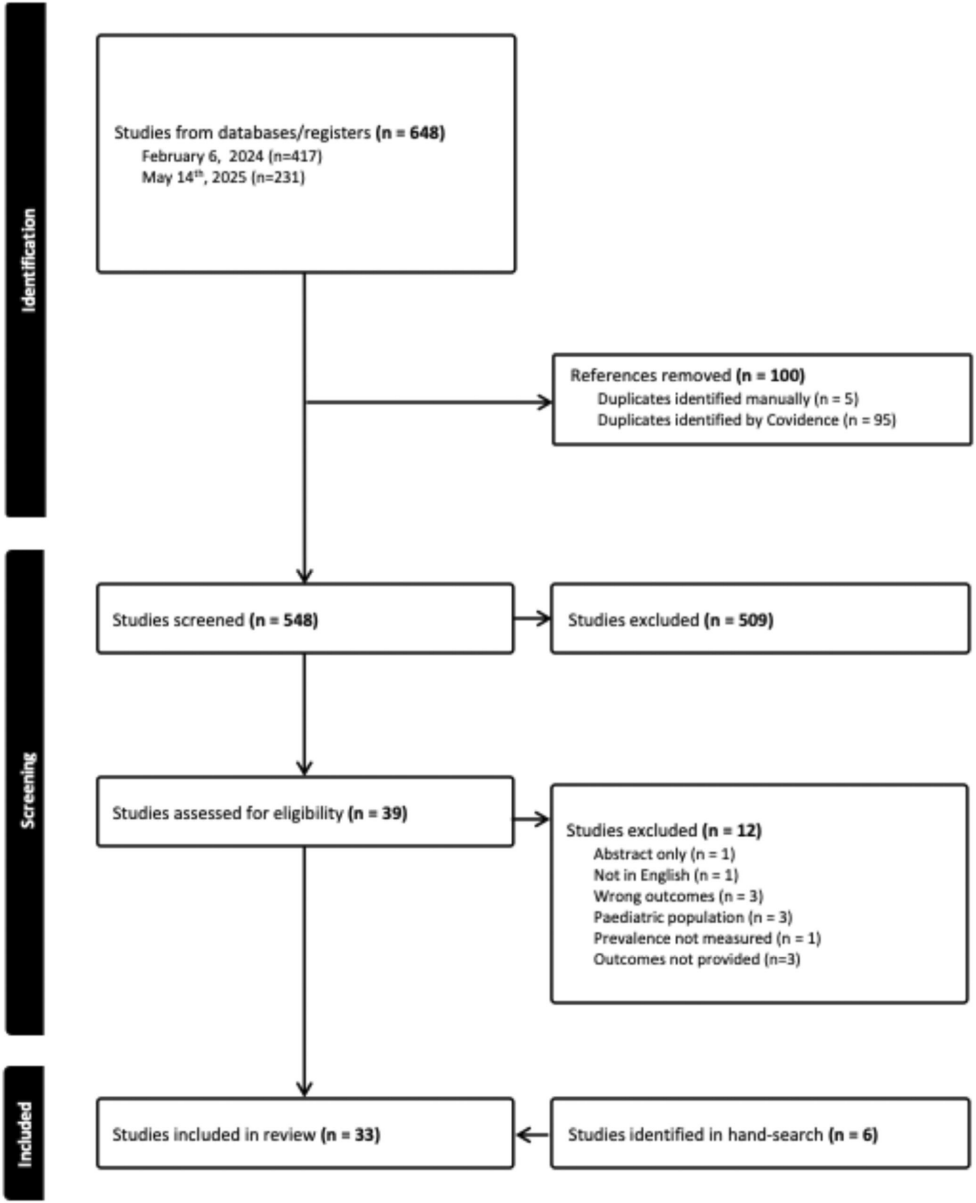
PRISMA diagram.

Retrieved studies were uploaded into Covidence^26^ web‐based software for systematic reviews. Duplicates were removed automatically, and two independent investigators (JNK, RDL) performed title and abstract screening followed by the full‐text screening. Disagreements were resolved by a third reviewer (AAD). Figure 1 depicts the PRISMA diagram detailing the study selection process. Interrater reliability was assessed by calculating a Cohens K.

### 2.4 Data extraction and quality assessment

Included studies underwent dual quality review by two independent reviewers (JNK, RDL) using the Newcastle‐Ottawa Scale (NOS) to evaluate risk of bias. Briefly, the NOS uses a star system to quantitatively assess study quality across three domains: (i.) the selection of the study groups, (ii.) the comparability of the groups and (iii.) the ascertainment of either the exposure or outcome of interest for case–control or cohort studies. Each of the 8 items can be awarded a star while comparability can be awarded two stars creating a 0–9 scale with higher scores indicating high quality.^27^, ^28^ Quality assessment conflicts were resolved by a 3rd reviewer (AAD).

### 2.5 Statistical analysis

The meta‐analysis was carried out in Stata. A random‐effects meta‐analysis of proportions was used to quantify the prevalence of new‐onset diabetes in COVID‐19. Prevalence, or proportional data, is based on a binomial distribution, specifically the ratio of “events” to “non‐events” within the sample. The Stata *metaprop* command was employed to account for the binomial distribution, asymmetric variances, and adjustment for asymmetric variances if necessary (using Freeman‐Tukey double arcsine transformation and stabilisation).^29^ A random‐effects model was chosen to account for the heterogeneity observed across studies in both the measurement of new‐onset diabetes and the populations studied. The prevalence of new‐onset diabetes was quantified overall by the sample reported in the studies. In addition to weighted estimates, the 95% confidence interval is reported along with z‐tests (weighted estimate divided by the standard error of the weighted estimate), and the associated p‐value was calculated. To describe a fraction of the variance due to heterogeneity *I* ^2^ was calculated. To further explain heterogeneity, a random‐effects meta‐regression was performed. The primary factors of interest were age, sex, and follow‐up duration (all at the study level). Predictor variables were examined for statistical significance using p‐values, the slope coefficient, and ± standard error. A *p*‐value ≤0.05 was considered significant in all models. Given the limited number of studies and variability in reporting, the analyses were considered exploratory. We acknowledge that study‐level meta‐regression may be underpowered to detect meaningful associations. Post hoc analyses were conducted to explore whether disease severity, using hospitalisation as a proxy, contributed to heterogeneity among the included studies.

## 3 RESULTS

The systematic literature search identified 648 articles for review. After removing duplicates, 548 articles underwent title and abstract screening. Twenty articles were identified for data extraction and analysis. To identify articles published in the interim since February 6th, 2024, a follow‐up search was conducted (May 14th, 2025) that identified 231 additional articles for title and abstract screening. Twelve articles underwent full‐text review, and seven articles were retained for data extraction and analysis. Last, a hand search was performed identifying six additional articles. In total, 33 articles were retained for analysis. Interrater reliability between the first and second coder, represented by Cohens k coefficient, was 0.57 indicating moderate agreement. In terms of quality assessment, all included studies were identified as “good” meaning high quality with low risk of bias according to the New Castle Ottawa Scale. A summary table with study characteristics and key findings is provided in the Supporting Information.

The included studies spanned 17 different countries (United States *n* = 8,^30^, ^31^, ^32^, ^33^, ^34^, ^35^, ^36^, ^37^ India *n* = 3,^38^, ^39^, ^40^ England *n* = 3,^41^, ^42^, ^43^ Ethiopia *n* = 2,^44^, ^45^ Italy *n* = 2,^46^, ^47^ United Kingdom *n* = 2, Australia *n* = 1,^48^ Bangladesh *n* = 1,^49^ China *n* = 1,^50^ Egypt *n* = 1,^51^ Germany *n* = 1,^52^ Korea *n* = 1,^53^ Romania *n* = 1,^54^ Saudi Arabia *n* = 1,^55^ South Africa *n* = 1,^56^ Sudan *n* = 1,^57^ United Arab Emirates n = 1^58^) and two studies spanned multiple countries.^59^, ^60^ Cumulatively, study collection time frames spanned from January 2017–July 2023 and included Alpha, Beta, Delta, Gamma and Omicron COVID‐19 variants. Study identifiers for new‐onset DM are described in Table 1. COVID‐19 infection identification methods are provided in Table 1. From the available data, slightly more men than women were included across studies (54.62 95% CI [54.6196, 54.6204]). Twenty one (21/33 63.6%) studies reported findings on new‐onset DM (combined T1DM and T2DM or unspecified),^32^, ^35^, ^37^, ^38^, ^39^, ^40^, ^42^, ^44^, ^45^, ^47^, ^48^, ^49^, ^51^, ^52^, ^54^, ^55^, ^56^, ^57^, ^58^, ^61^, ^62^ eleven studies (11/33 33%) reported on new onset T2DM^30^, ^33^, ^35^, ^43^, ^44^, ^46^, ^50^, ^52^, ^53^, ^59^, ^60^ and seven studies (7/33 21.2%) reported on new onset T1DM.^31^, ^34^, ^35^, ^36^, ^41^, ^44^, ^59^ Studies that evaluated T1DM and T2DM independently were classified within their respective individual category as well as the combined category.

**TABLE 1:** Study identifiers for new‐onset diabetes and COVID‐19.

Study identifiers for diabetes	
American Diabetes Association diagnostic criteria (haemoglobin A1c (HbA1c) ≥6.5, fasting plasma glucose (FPG) ≥126 mg/dL, Oral glucose tolerance test (OGTT ≥200 mg/dL)	37, 39, 44, 45, 47, 48, 49, 50, 51, 54, 55, 61
ICD‐9/10 codes	31, 32, 33, 34, 35, 36, 43, 46, 52, 59, 60
HbA1c >6.4	38
Indian Council of Medical Research (ICMR) criteria (FPG ≥126 mg/dL, OGTT ≥200, HbA1c ≥6.5)	40
No history of DM, no DM prescription medications, and FPG during hospitalisation	47
Health records	53, 58
Not provided	30, 41, 42, 56, 57

Study identifiers for COVID‐19	
Reverse transcriptase polymerase chain reaction (RT‐PCR)	30, 31, 32, 44, 45, 49, 54, 55, 57, 58
Clinical manifestations of COVID‐19 and RT‐PCR	39, 50
Positive for SARS‐CoV‐2 RNA	51
Systematised Nomenclature of Medicine—Clinical Terms (SNOMED‐CT), RT‐PCR or lateral flow antigen test of SARS‐CoV‐2	41
RT‐PCR and antigen testing	38, 56
ICD‐10 codes	33, 34, 42, 46, 52, 53, 59, 60
RT‐PCR and radiologic confirmation	40, 47
RT‐PCR or clinical diagnoses of confirmed or suspected COVID‐19	35, 62
ICD‐10 code or positive COVID‐19 test	36, 37
Medical record	48
Not reported	43

### 3.1 Meta‐analysis

**FIGURE 2:**
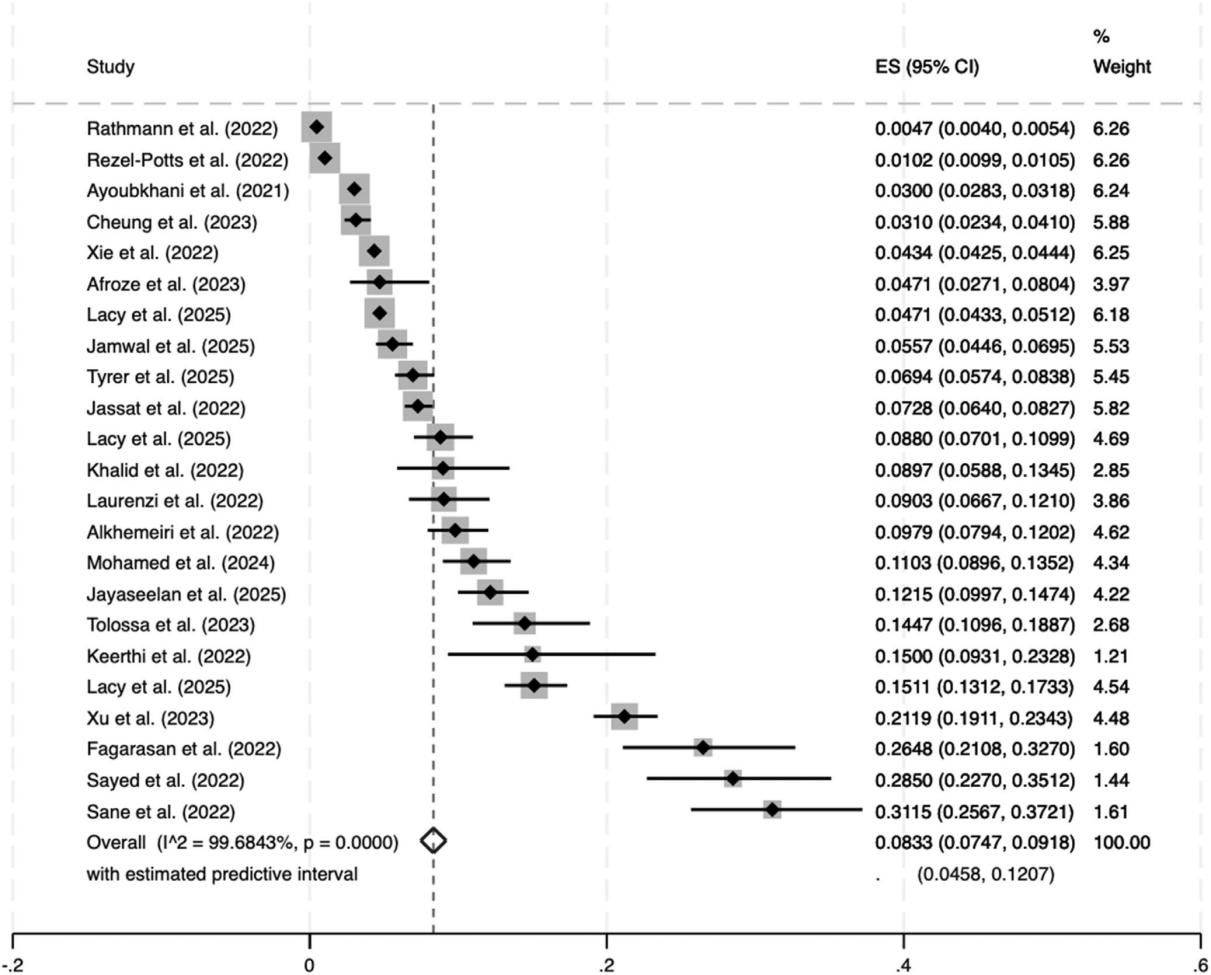
Estimated combined (T1DM, T2DM, or undefined) prevalence of new‐onset diabetes after SARS‐CoV‐2 infection.

**FIGURE 3:**
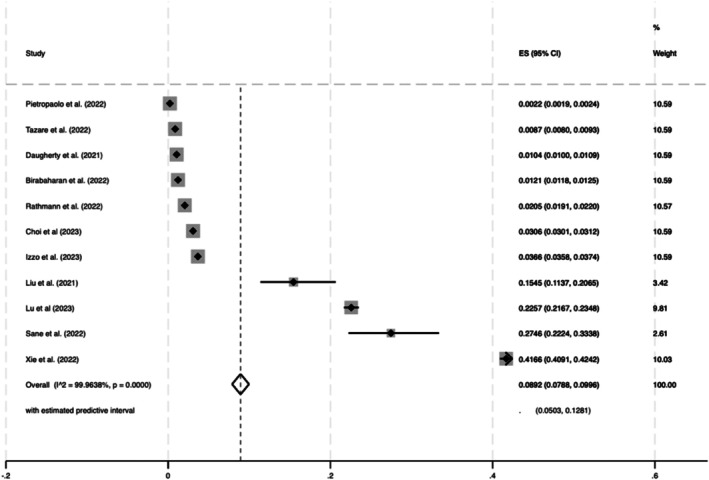
Estimated prevalence of T2DM after SARS‐CoV‐2 infection.

**FIGURE 4:**
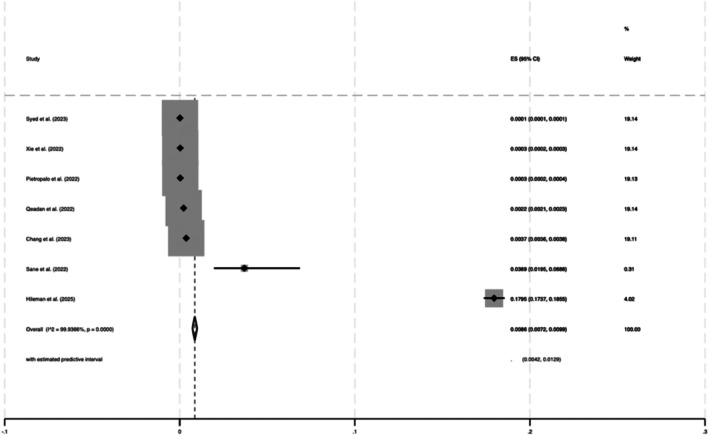
Estimated prevalence of T1DM after SARS‐CoV‐2 infection.

The overall estimated prevalence of new‐onset diabetes (combined T1DM and T2DM or undefined) following COVID‐19 infection was 8.33% (95% CI 7.47, 9.18% z = 19.04, *p* < 0.001; Q = 6791.24, *I* ^2^, 99.68%) (Figure 2). The overall estimated prevalence of new‐onset T2DM in COVID‐19 was slightly higher 8.92% (95% CI 7.88%, 9.96%, z = 16.77, *p* < 0.001; *Q* = 27659.74; *p* < 0.001, *I* ^2^ = 99.96%) (Figure 3). The overall estimated prevalence of new‐onset T1DM following COVID‐19 was 0.86% (95% CI 0.0072%, 0.0099%, z = 12.59, *p* < 0.001; *Q* = 9456.28; *p* < 0.001, *I* ^2^ = 99.94%) (Figure 4).

### 3.2 Meta‐regression

Based on our a priori analytical plan, we examined age, sex, and follow‐up duration as predictor variables in the prevalence of new‐onset diabetes at the study level (Supporting Information). There was no significant relationship with age at the study level (β = 0.0025, t = 1.37, *p* = 0.186), meaning that studies tending to include older people did not report different rates of new‐onset diabetes prevalence. Similarly, no significant relationship was found for sex (β = −0.045, t = −0.28, *p* = 0.779), indicating that at the study level, no differences were observed in the prevalence of new‐onset diabetes in studies that had a greater proportion of men versus women. Importantly, no significant relationship was identified for follow‐up duration (β = 0.00041, t = −0.25, *p* = 0.809). Thus, there was no difference in the prevalence of new‐onset diabetes identified in studies with longer follow‐up times (up to 3 years).

### 3.3 Post hoc analyses

Given high heterogeneity observed, we conducted post hoc analyses. The overall estimated prevalence of new‐onset diabetes (combined T1DM and T2DM or undefined) following COVID‐19 infection in hospitalised patients is 7.93% (95% CI 6.92, 8.95% z = 15.35, *p* ≤ 0.001; Q = 6224.62, *I* ^2^, 99.76%), non‐hospitalised patients 4.71% (95% CI 2.71, 8.04% z = 3.55, *p* ≤ 0.001) and both (hospitalised and non‐hospitalised) 9.70% (95% CI 6.39, 13.02% z = 5.73, *p* ≤ 0.001; Q = 6968.19, *I* ^2^, 96.93%). Post hoc analyses for T1DM and T2DM in hospitalised patients yielded limited scientific insight due to comparing single studies and very small groupings of studies. These analyses are provided in the Supporting Information.

## 4 DISCUSSION

This systematic review and meta‐analysis aimed to derive a precise estimate of the prevalence of new‐onset diabetes in patients with COVID‐19 based on data from 33 studies involving 53 211 patients globally. We identified similar rates for new onset T2DM (9%) and combined T1DM and T2DM or undefined (8%). However, the rate of new onset T1DM was relatively low (1.0%). At the study level, the prevalence of new‐onset diabetes is not a function of age, sex, or follow up duration. However, these analyses are limited by the small number of included studies and the heterogeneity of study design. As such, results should be interpreted cautiously and further research using individual‐patient level data may be needed to investigate potential sources of heterogeneity.

To contextualise the impact of COVID‐19 on diabetes prevalence, baseline rates in the general population merit consideration. As of 2022, approximately 14% of adults aged 18 years and older were living with diabetes globally, representing a twofold increase from 7% in 1990.^63^ This background prevalence is similar to the pooled diabetes prevalence of 14.7% (95% CI 12.5–16.9%) reported in a meta‐analysis of 729 studies involving nearly 30 million COVID‐19 patients.^64^ In addition, rates are higher among hospitalised individuals (21.4%) and up to 28.9% among severe COVID‐19 cases. This study includes data up to May of 2022. Herein, we analyse an additional 3 years of data and applied more stringent inclusion criteria to select higher quality studies for analysis.

Importantly, few studies distinguished between T1DM^31^, ^34^, ^35^, ^36^, ^41^, ^44^, ^59^ and T2DM.^30^, ^33^, ^43^, ^44^, ^46^, ^50^, ^52^, ^53^, ^59^, ^60^ The finding that T2DM has a higher prevalence could be because of the population demographic (i.e., adults ≥18 years) or the fact that 90–95% of people with diabetes globally have T2DM.^65^ The increased risk for T2DM diabetes in older adults has been well established.^66^ The mean patient age in our study is 50.32 years (range: 34–69 years, IQR 20.5). The risk of T2DM increases dramatically after the age of 55 years—yet earlier onset (≤ 40 years) is rising.^66^ A study comprising 11 cohorts with 47.1 million participants examined individuals of all ages (with and without COVID‐19) reported similar findings for risk of T2DM (RR = 1.78; 95% CI, 1.56–2.02) that exceeded risk of T1DM (RR = 1.42; 95% CI 1.38–1.46).^13^ Importantly, no significant differences in risk were observed across age groups.^13^

Notably, the incidence T1DM has more frequently been cited for paediatric (<18 years) populations. Although T1DM can be diagnosed at any age, peak age for T1DM diagnosis is between 5 and 7 years and again around the onset of puberty.^67^ Increased risk of developing T1DM post viral infection (i.e., coxsackie, echovirus, rubella, cytomegalovirus, mumps, and rotavirus) has been reported.^68^ As an autoimmune disorder, T1DM aetiology includes both strong genetic and environmental contributions underlying immune mediated ß cell dysfunction requiring lifelong insulin dependence.^69^ A triggering insult (i.e., exposure viruses) can initiate this process.^69^ From 2019 to 2020, the global incidence of T1DM increased from 19.73 to 32.39 per 100 000.^70^ Data show a 9.5% increase in new‐onset paediatric T1DM cases compared with the pre‐pandemic period.^70^ A meta‐analysis of 17 studies including 38 149 children revealed a significantly higher incidence rate during the first year of the COVID‐19 pandemic compared with the pre‐pandemic period (IRR, 1.27; 95% CI, 1.18–1.37).^71^

In contrast to T1DM, T2DM is characterised by insulin resistance and associated ß cell dysfunction.^72^ Type 2 DM is commonly seen in individuals >55 years—yet may occurs at any age.^72^ It is more common in individuals who are obese with greater fat mass.^73^ Obesity induces a chronic pro‐inflammatory state contributing insulin resistance and the pathogenesis of T2DM.^74^ As COVID‐19 induces a robust inflammatory response,^75^ which can disrupt lipid metabolism, increasing insulin resistance and potentially mediating the development of T2DM.^76^ In particular, β cells are susceptible to infection via known SARS‐CoV‐2 viral entry factors angiotensin‐converting enzyme (ACE2) and transmembrane protease, serine 2 (TMPRSS2), and dipeptidyl peptidase‐4 (DPP4).^77^ Notably, β cells have been associated with de‐differentiation or trans‐differentiation, degranulation, cellular injury and necroptosis.^77^, ^78^, ^79^ Similarly, adipocytes are susceptible to infection acting as a viral reservoir that gives rise to proinflammatory cytokines and infiltrating macrophages resulting in insulin resistance.^76^ Moreover, acute severe illness resulting in lipolysis gives rise to gluconeogenesis, which may also be contributing to hyperglycemia.^76^

The prevalence of T2DM observed in our study is lower than those reported in prior meta‐analyses, ranging from 16% to 20%.^18^, ^23^, ^80^ A meta‐analysis of 20 studies with 320 948 participants reported a pooled diabetes prevalence of 16% (95% CI: 11–22%)^23^—a rate that was likely influenced by the broader inclusion criteria (including no age restrictions). Another meta‐analysis focusing specifically on hospitalised COVID‐19 patients found a higher weighted prevalence of 20% (95% CI: 15.0–25.0; *I* ^2^ = 99.3%)^80^—yet findings may reflect the increased disease severity of inpatients and likely concomitant heightened systemic inflammation in those hospitalised for severe COVID‐19.

An increased incidence of diabetes during the COVID‐19 pandemic has been previously reported in large population‐based studies. An Italian study comparing pre‐pandemic data (2017–2019) to the pandemic period (2020–2022) found T2DM incidence increased from 4.85 (95% CI: 4.68–5.02) to 12.21 (95% CI: 11.94–12.48) per 1000 person‐years.^46^ Consistent with these data, a large epidemiologic study comprised of 10 cohorts and 40 million participants of all ages examined the relative risk of diabetes after COVID‐19 infection.^24^ Investigators reported an overall incidence of 15.53 (95% CI: 7.91–25.64) per 1000 person‐years, with increased relative risk for both T2DM (RR = 1.70; 95% CI: 1.32–2.19) and T1DM (RR = 1.48; 95% CI: 1.26–1.75) compared with non‐infected individuals.^24^ Similarly, a meta‐analysis of 8 studies including over 4.2 million patients found a 66% increase in risk of new‐onset diabetes after COVID‐19 infection (RR = 1.66; 95% CI: 1.38–2.00).^81^ The study provides foundational knowledge on new‐onset diabetes across age groups (up to October 2022) across three countries (United States, United Kingdom, and Germany). The present study extends prior findings by examining the literature over a longer duration with a more granular focus on adult populations.

While the mechanism underlying metabolic disturbance with COVID‐19 has yet to be elucidated, it is likely due to several underlying factors. Proposed mechanisms include altered glucose production, glucose metabolism and/or insulin secretion, previously undiagnosed diabetes,^12^, ^82^ steroid‐induced diabetes,^12^ destruction of islet ß cells,^82^, ^83^ and adipose tissue dysfunction.^76^, ^82^ Well‐designed prospective studies are needed to better elucidate the underlying mechanisms and improve our understanding of the true prevalence of new‐onset diabetes. Specifically, such work should carefully control for potential misclassification, including previously undiagnosed diabetes and transient steroid or stress induced hyperglycemia. It is plausible that underlying genetic risk and social determinants of health (SDoH) interact and may play a role in metabolic dysfunction. The development of DM is multifactorial and influenced by both genetic and environmental factors.^72^, ^84^ During the COVID‐19 pandemic, social distancing and changes in environmental conditions contributed to reduced physical activity and increased consumption of unhealthy food choices.^85^ Such lifestyle changes contributed to adverse cardiometabolic risk factors.^86^ Resulting social and environmental changes were further compounded by disrupted healthcare delivery services, reduced health seeking behaviours, and ultimately fewer interactions with healthcare providers.^87^

As gene–environment interactions strongly influence the pathogenesis of both T1DM and T2DM, lifestyle, exercise, and diet are all key drivers in T2DM development.^72^ Future research should investigate social and environmental factors and underlying genetic and epigenetic factors that may contribute to new onset DM following COVID‐19 infection.

The findings from this study differ from previously published comparative studies that examined the risk of diabetes relative to uninfected populations^13^, ^24^, ^81^ or pre‐pandemic periods.^70^, ^71^ Herein, we report real‐world incidence estimates that reflect variation across patient populations and clinical practice settings. Our findings can inform clinical decision making across heterogeneous healthcare environments. It is important for clinicians to appreciate the potential metabolic sequalae of COVID‐19 and consider targeted screening—particularly for patients with increased risk related to severe infection/hospitalisation and/or pre‐existing cardiometabolic risk. Threshold‐based diagnostic testing, including haemoglobin A1c (HbA1c) and fasting glucose measurements, may facilitate early identification of dysglycemia and timely management to mitigate long‐term complications. Increased awareness of post‐COVID‐19 metabolic risk can help guide patient monitoring and optimise clinical outcomes for patients.

## 5 STRENGTHS AND LIMITATIONS

Relative strengths of this review and meta‐analysis include a comprehensive review of the literature and utilised a rigorous dual review process and a well‐established framework to inform potential bias.^28^ It is important to note this work has several limitations. Despite every effort being made to identify completed studies on new‐onset diabetes in COVID‐19 patients, it is possible that we inadvertently missed research in this area. We know virulence differed across COVID‐19 variants; this was not examined as many studies did not report prominent variant type. Outcomes may be influenced by comorbidities and medication use (i.e., corticosteroids a common treatment for COVID‐19) or lifestyle factors that were not analysed. Importantly, definitions of new‐onset diabetes relied on varying criteria (Table 1). As a result, transient or stress‐induced hyperglycemia as well as undiagnosed diabetes may have been misclassified as true new‐onset diabetes.

## 6 CONCLUSION

Synthesising the findings from the systematic review and meta‐analysis reveals an increased prevalence of DM (T2DM or combined T1DM and T2DM or undefined) among adults after SARS‐CoV‐2 infection. Data also suggest minimal change in prevalence of T1DM among adults infected with SARS‐CoV‐2. Considering the evolving nature of COVID‐19 and the emergence of new variants, it is important to gain a better understanding of the underlying factors contributing to the increased prevalence.

## FUNDING INFORMATION

This study was supported by National Institute of Health, National Institute of Nursing Research, 1F31NR021624‐01.

## CONFLICT OF INTEREST STATEMENT

The authors declare no conflicts of interest.

## Supporting information


**Data S1:** Supporting Information.

**Data S2:** Supporting Information.

## Data Availability

The data that support the findings of this study are available in the Supporting Information of this article.
