# Supplementary material for: Prevalence of new‐onset diabetes following COVID‐19 infection: A systematic review and meta‐analysis
Source: Diabetes Obes Metab. 2026 Jan 28;28(4):3182–92. doi: 10.1111/dom.70508 (PMC12992178; doi:10.1111/dom.70508)

**Association between age and new-onset diabetes**
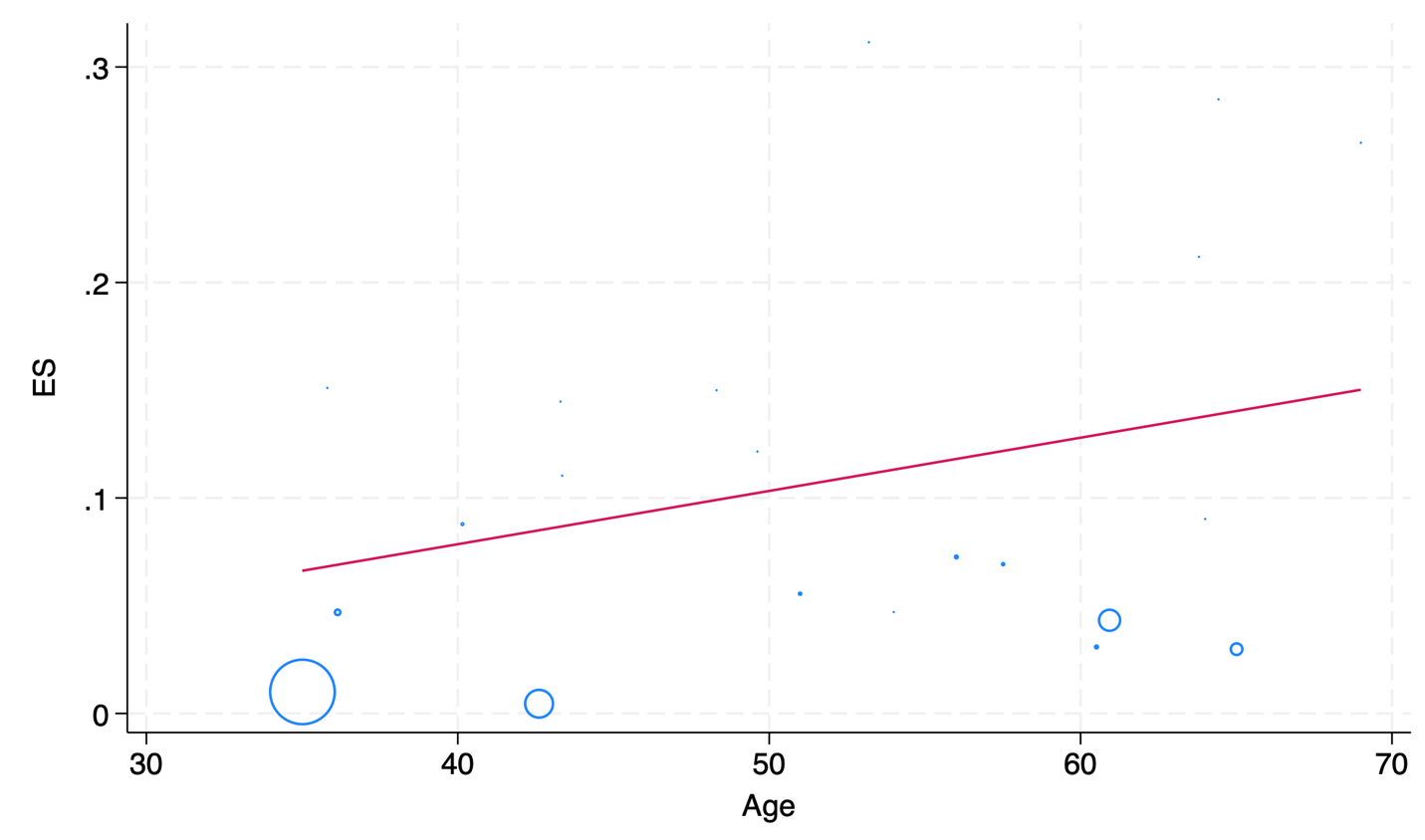


**Association between gender and new-onset diabetes**


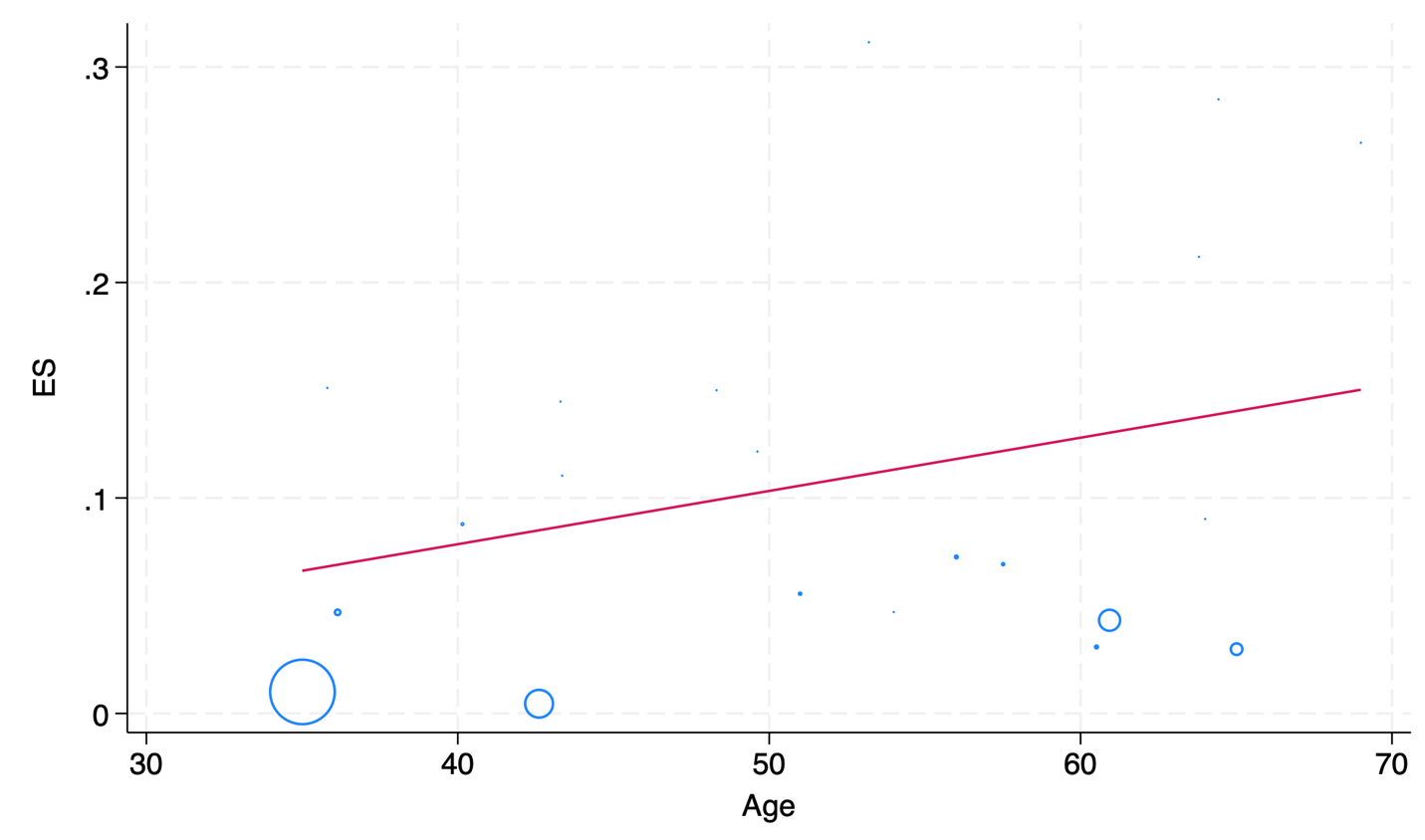


**Association between follow-up duration and new-onset diabetes**


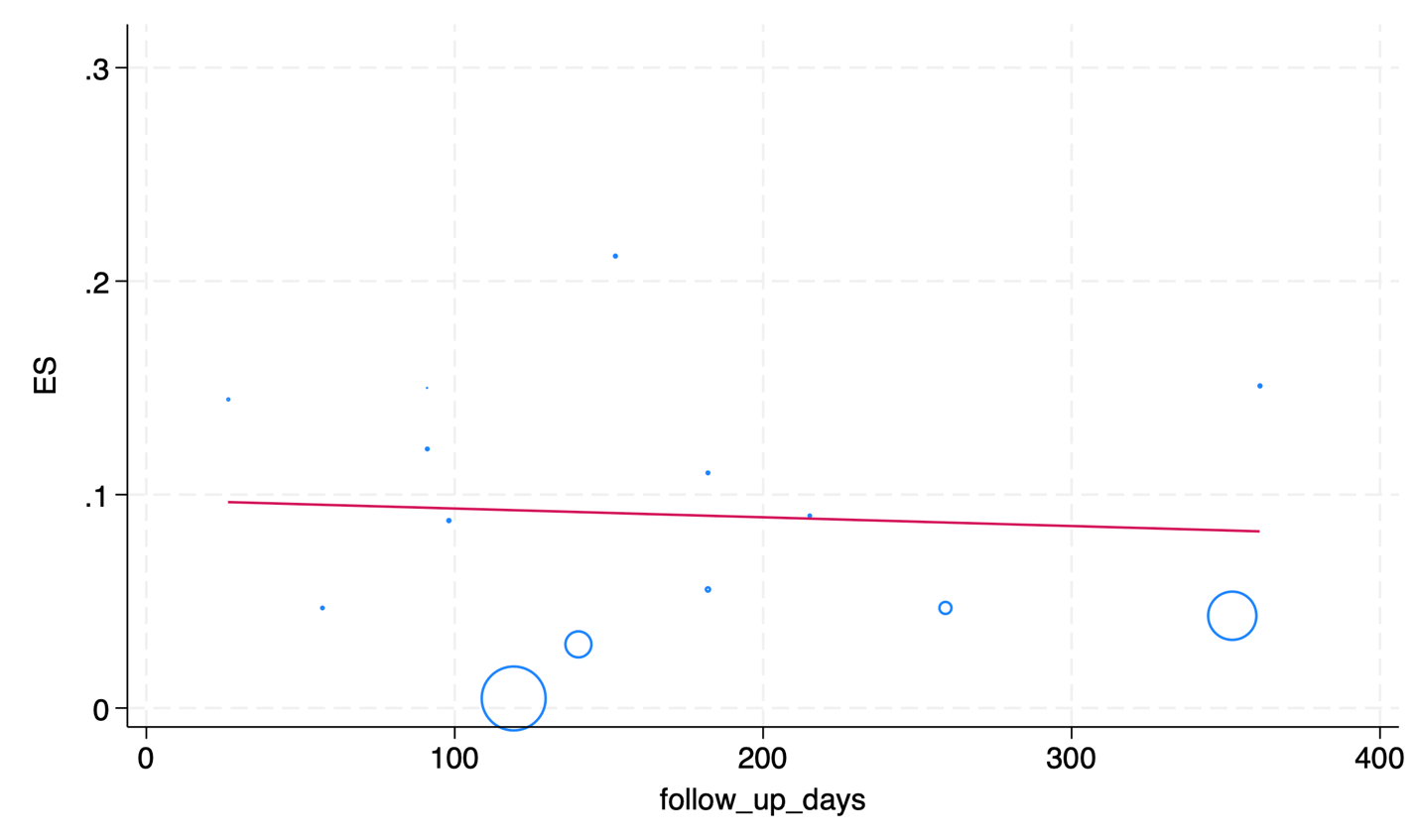


**Estimated prevalence of new-onset diabetes after SARS-CoV-2 infection by hospitalization status.**


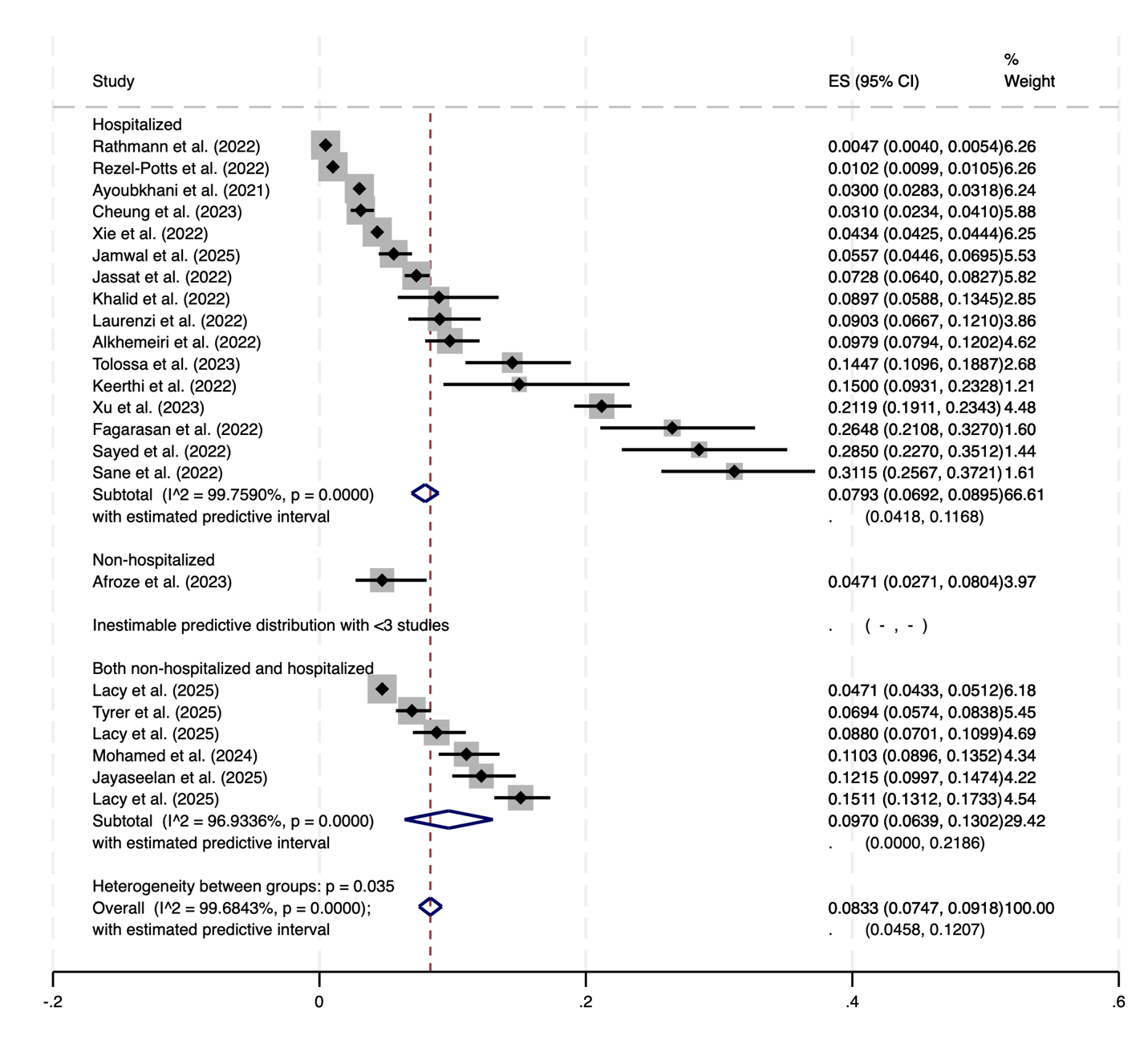


**Estimated prevalence of new-onset diabetes T2DM after SARS-CoV-2 infection by hospitalization status.**


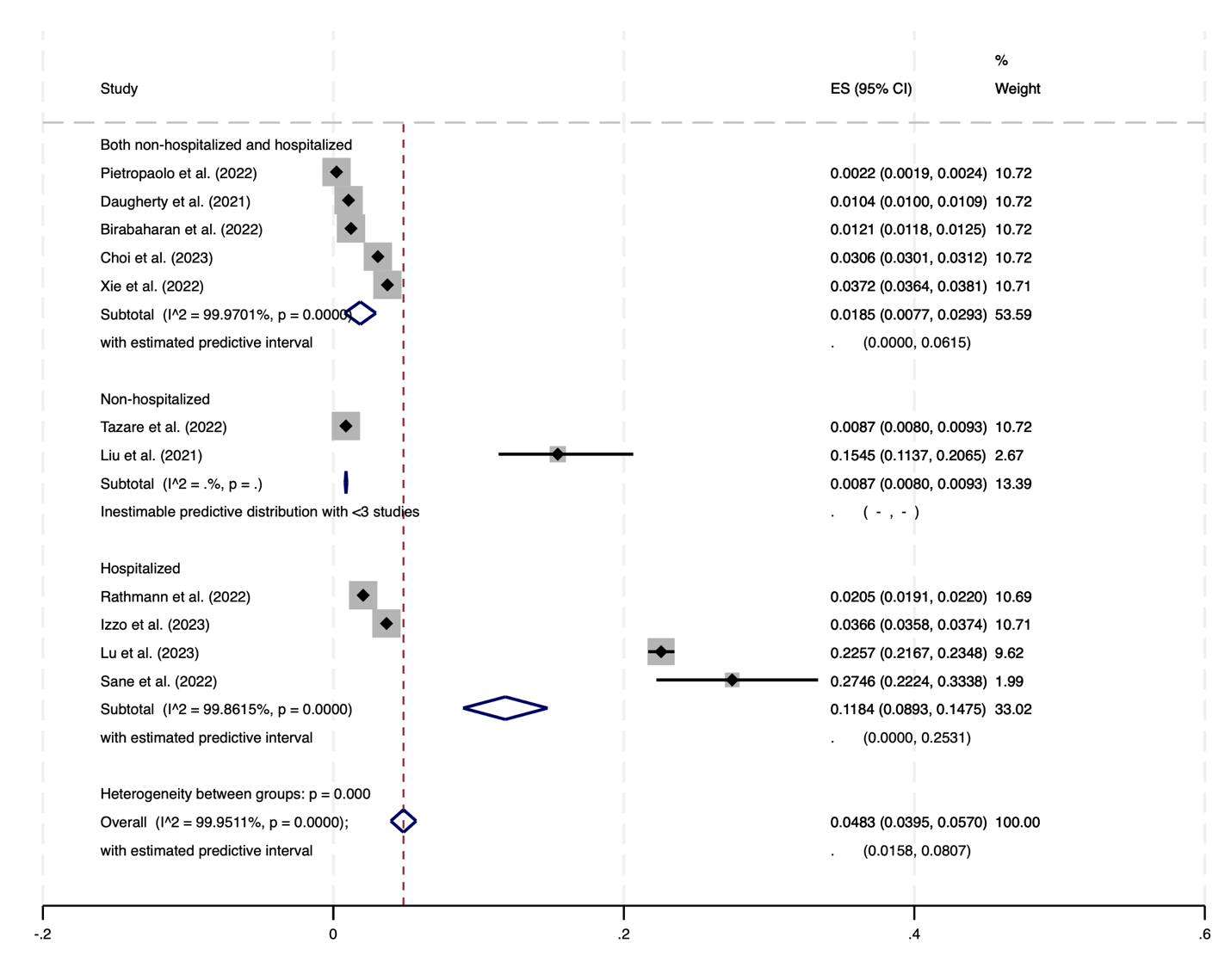

Supplement: Supplementary file 1 — Data S1: Supporting Information. [file DOM-28-3182-s002.docx]
